# School Water, Sanitation, and Hygiene, Soil-Transmitted Helminths, and Schistosomes: National Mapping in Ethiopia

**DOI:** 10.1371/journal.pntd.0004515

**Published:** 2016-03-08

**Authors:** Jack E. T. Grimes, Gemechu Tadesse, Kalkidan Mekete, Yonas Wuletaw, Abeba Gebretsadik, Michael D. French, Wendy E. Harrison, Lesley J. Drake, Iain A. Gardiner, Elodie Yard, Michael R. Templeton

**Affiliations:** 1 Department of Civil and Environmental Engineering, South Kensington Campus, Imperial College London, London, United Kingdom; 2 Ethiopian Public Health Institute, Addis Ababa, Ethiopia; 3 Schistosomiasis Control Initiative, Department of Infectious Disease Epidemiology, St Mary’s Campus, Imperial College London, London, United Kingdom; 4 Partnership for Child Development, Department of Infectious Disease Epidemiology, St Mary’s Campus, Imperial College London, London, United Kingdom; Swiss Tropical and Public Health Institute, SWITZERLAND

## Abstract

**Background:**

It is thought that improving water, sanitation, and hygiene (WASH) might reduce the transmission of schistosomes and soil-transmitted helminths, owing to their life cycles. However, few large-scale studies have yet assessed the real extent of associations between WASH and these parasites.

**Methodology/Principal Findings:**

In the 2013–2014 Ethiopian national mapping of infections with these parasites, school WASH was assessed alongside infection intensity in children, mostly between 10 and 15 years of age. Scores were constructed reflecting exposure to schistosomes arising from water collection for schools, from freshwater sources, and the adequacy of school sanitation and hygiene facilities. Kendall’s *τ*_*b*_ was used to test the WASH scores against the school-level arithmetic mean intensity of infection with each parasite, in schools with at least one child positive for the parasite in question.

WASH and parasitology data were available for 1,645 schools. More frequent collection of water for schools, from open freshwater sources was associated with statistically significantly higher *Schistosoma mansoni* infection intensity (Kendall’s *τ*_*b*_ = 0.097, 95% confidence interval, CI: 0.011 to 0.18), better sanitation was associated with significantly lower *Ascaris lumbricoides* intensity (Kendall’s *τ*_*b*_ = -0.067, 95% CI: -0.11 to -0.023) and borderline significant lower hookworm intensity (Kendall’s *τ*_*b*_ = -0.039, 95% CI: -0.090 to 0.012, *P* = 0.067), and better hygiene was associated with significantly lower hookworm intensity (Kendall’s *τ*_*b*_ = -0.076, 95% CI: -0.13 to -0.020). However, no significant differences were observed when comparing sanitation and infection with *S*. *mansoni* or *Trichuris trichiura*, or hygiene and infection with *A*. *lumbricoides* or *T*. *trichiura*.

**Conclusions/Significance:**

Improving school WASH may reduce transmission of these parasites. However, different forms of WASH appear to have different effects on infection with the various parasites, with our analysis finding the strongest associations between water and *S*. *mansoni*, sanitation and *A*. *lumbricoides*, and hygiene and hookworm.

## Author Summary

Water, sanitation, and hygiene (WASH) are potentially important control measures for parasitic worms, but few large-scale and well-designed research studies have aimed to quantify the relationships between WASH and infections with these parasites, or demonstrated the practicality of integrating WASH assessments into large-scale worm mapping. Here we use data from 80,475 children in 1,645 schools visited during the 2013–2014 Ethiopian national mapping of schistosomiasis and soil-transmitted helminthiases, to compare school WASH conditions with the mean intensity of infection with these parasites, using Kendall’s *τ*_*b*_, a statistic that assesses for correlation between two variables by comparing their rankings. More frequent collection of water for schools was associated with statistically significantly (*P* < 0.05) higher-intensity intestinal schistosomiasis infections, better sanitation significantly with lower roundworm burdens, and better hygiene with significantly lower hookworm burdens. No statistically significant associations (*P* > 0.05) were found for sanitation and intestinal schistosomiasis or whipworm infections, or hygiene and roundworm or whipworm infections, but better sanitation was associated with a borderline statistically significantly less hookworm infections (*P* = 0.067). The results suggest that WASH improvements might have different impacts on infection with the various parasites. These differences should be borne in mind when designing WASH interventions to reduce transmission of, and prevent infection with, these parasites.

## Introduction

Improvements in child health and education are key targets in many international development programs, but relatively little is known about the multiple health and educational benefits of integrating different school-based health interventions. Preventive chemotherapy (PC) can reduce morbidity caused by schistosome and soil-transmitted helminth (STH) infections [1–3], while improvements in water, sanitation, and hygiene (WASH) in schools might improve attendance and educational attainment [4]. But might WASH also reduce the transmission of schistosomes and STHs, and thus slow reinfection following PC? Answering such questions, and developing appropriate, sustainable, and scalable school health interventions, is likely crucial to the control of these parasites and to improve the health and education of children in low-income countries.

The transmission of schistosome (including *Schistosoma mansoni*) and STH (*Ascaris lumbricoides*, *Trichuris trichiura*, and the hookworms: *Ancylostoma duodenale* and *Necator americanus*) infections might be disrupted with WASH improvements, given the nature of their life cycles [1–3,5]. Safe water supplies might be defined as being free of the parasite eggs or larvae, and should reduce exposure to these parasitic infections. Adequate sanitation could be defined as that which contains feces harboring eggs until their larvae are inactivated, thus preventing contamination of the environment and subsequent infections of humans or intermediate host snails. STH eggs are not immediately infective, needing time on the order of days to embryonate and become infective [6], so an infected person is unlikely to become superinfected by ingesting eggs he or she has just excreted. However, when visiting a latrine or an area in the school used for defecation, he or she might touch the ground or walls, thus picking up and later potentially ingesting embryonated eggs previously left there. Washing the hands after visiting these areas might help to remove these eggs and thus to prevent infection.

People with access to adequate sanitation at home have statistically significantly lower (*P* < 0.05) odds of infection with *S*. *mansoni*, *A*. *lumbricoides*, and *T*. *trichiura* [7–9]. Regarding hookworm infection and sanitation, one meta-analysis found a significant association [7], while another did not [9]. Handwashing after defecation was associated with significantly lower odds of infection with any STH [9], and safe water supplies with significantly lower odds of schistosome infection [8]. However, as mentioned in these reviews, socioeconomic status (SES) may confound the relationship between WASH and infection. People of higher SES are likely to have better household WASH, but are also likely protected from infection by a number of other factors including lower occupational exposure, better knowledge of the parasite transmission, and better access to treatment.

In Ethiopia, school WASH is generally provided by the government or by non-governmental organizations (NGOs)–it is therefore less reflective of its users’ SES. Schoolchildren spend a substantial portion of the waking day in school, suggesting that the school environment may play an important role in the transmission of schistosomes and STHs. This particularly applies to the STHs, which do not rely upon intermediate host snails for transmission. Furthermore, large-scale helminth control programs are frequently directed at children, and use schools for the assessment of baseline levels of infection, for PC, and for monitoring and evaluation [10]. Integrating school WASH assessments into these school-based activities requires less time and is therefore more feasible than integrating the assessment of WASH in households or other places.

In the 2013–2014 Ethiopian national mapping of schistosomes and STHs, WASH was assessed alongside the parasite infections in each school selected for the survey. Data were available for 80,475 children in 1,645 schools. This paper presents for the first time a national-scale school-level analysis comparing schools’ water, sanitation, and hygiene with their parasitic infection intensities, while more details of the national distributions of these parasites will be presented elsewhere. The motivation of this analysis was to investigate quantitatively whether schools with better WASH had lower rates of parasitic infection, which would advocate strongly for school WASH to be included as an essential element of reducing transmission.

## Methods

### Ethical considerations

Ethical approval for the national mapping project was granted by the Scientific and Ethical Review Committee of the Ethiopian Public Health Institute. On the Imperial College side, the project was covered by ethical approval granted by Imperial College Research Ethics Committee to the Schistosomiasis Control Initiative for surveillance and monitoring activities for the evaluation of national control programs for control of neglected tropical diseases (reference: ICREC_8_2_2). Informed written consent was sought from each school director in place of the parents, while informed verbal consent was sought from each child providing samples and receiving anthelmintic treatment. All students tested were treated with 400 mg of albendazole, while all positive schistosomiasis cases were treated with 40 mg/kg body weight of praziquantel, using height measured against a tablet pole as a proxy [10].

### Study area and population

This study was carried out in primary schools across Ethiopia, which is one of the least urbanized countries in the world. Only around 16% of Ethiopians live in urban areas, and agriculture accounts for around 43% of the country’s gross domestic product [11]. In 2007, it was estimated that Ethiopia had a population of 73.8 million (and a population density of 67.1 people per km^2^) [11]. About half (52.1%) of Ethiopia’s female population, and 38.3% of its male population, have had no formal education. However, in the younger age groups, these figures are much lower [11], and around half of Ethiopians are below 15 years of age [11].

Schistosomes and STHs constitute serious public health problems in Ethiopia, with estimated nationwide prevalences of 16.5% and 28.8%, respectively [12,13]. Before the national mapping reported here, only limited schistosome monitoring and control had taken place in Ethiopia: in a review of neglected tropical diseases in Ethiopia, Deribe *et al*. (2012) report on a national survey that took place in 1988–1989 [14]. On the other hand, between the years of 2004 and 2009, more than 11 million preschool-age children (aged two to five years) received PC against STH infections [14].

Ethiopia has seen rapid improvements in household WASH in recent years. Between 1990 and 2015, the estimated proportion of the population practicing open defecation fell from 92% to 29%, and the estimated proportion without access to improved water supplies fell from 87% to 43% [15]. However, the above figures reflect substantial proportions of the population that still lack access to safe water supplies and adequate sanitation. Furthermore, improved water sources may only be available at long distances (e.g. several hundred meters or more) from the home for many people. Appropriately constructed sanitation facilities, particularly in schools, may have unacceptable hygienic conditions such as very unclean floors and walls, highly intolerable odors, and many flies.

### School selection

Schools were selected purposively at the woreda (district) level in most regions, with the exceptions of Afar and Somali, where zonal-level selection was employed because of the expectation of lower infection rates. Under purposive sampling, 10 schools per woreda were selected randomly. Next, five of those 10 schools were selected by the data collectors in consultation with the woreda health office, giving priority to schools thought to harbor the highest schistosome infection rates, given the local medical records and the presence of freshwater bodies. Amhara region was excluded from this mapping, to avoid duplication of activities of the Carter Center, who were mapping it for *S*. *mansoni* and STH infections.

### Field procedures

Data were collected by 134 laboratory technicians and health officers recruited from health offices in each region, and trained centrally at a four-day workshop on the parasitological techniques and WASH survey. Fifty students per school (approximately 25 boys and 25 girls, of ages roughly 10–15 years) were randomly selected for parasitological analysis. This randomization was usually effected by asking the children from grade five to stand in two lines: one for boys, and another for girls. Then, 25 children were selected at equal intervals along each line. In schools with fewer than 25 boys or girls in grade five, children from grades four and/or six were also included.

Each student provided one own stool sample, and one own urine sample. Urine samples were tested for hematuria at the school, using Hemastix reagent strips (Bayer HealthCare LLC, Elkhart, Indiana, USA). Hemastix-negative samples were considered negative for *S*. *haematobium*. From the Hemastix-positive urine samples, 10 ml was extracted and passed through filter paper of pore size 25 μm (Sefar MEDIFAB Polyamide 03-25/19, manufactured by Sefar AG, Heiden, Switzerland).

Concurrently, a health officer assessed the school’s WASH facilities and practices through a closed-ended questionnaire. This questionnaire combined inspections of the latrines and on-site water sources, with questions to the school director (head teacher), regarding the school’s WASH practices.

### Laboratory procedures

The stool samples, and filter papers through which Hemastix-positive urine samples had passed, were taken to the laboratory of the local woreda health office. There, the trained laboratory technicians employed the Kato-Katz method [16] on stool samples to prepare and examine one slide per participant for the enumeration of *S*. *mansoni* and STH eggs. Processing the samples at the health facility, then immediately reading the slides, minimized the risk of the hookworm eggs disappearing before the slides were prepared and examined [17,18].

The laboratory technicians also examined the filter paper microscopically, to quantify the *S*. *haematobium* eggs per 10 ml of urine sample. Samples positive for *S*. *haematobium* were defined as those that both (i) tested positive for hematuria according to Hemastix, and then (ii) exhibited *S*. *haematobium* eggs under microscopic examination.

### Data handling

Parasitological data were entered into smartphones and uploaded to a central server, using the LINKS system developed by the Task Force for Global Health, Atlanta, GA, USA. WASH data were collected on paper and subsequently entered into the Census and Survey Processing System (CS-Pro) version 5 (United States Census Bureau, Washington, DC, USA). In order to ensure the reliability of this data entry, the clerks were made aware that data for a 10% random sample of schools would be double-entered, and discrepancies reviewed. This double-entry revealed some discrepancies, resulting from data input errors, but no systematic errors characteristic of the entering of random data. Errors revealed in this manner were corrected by referring to the paper questionnaires. Subsequently, CS-Pro and SPSS versions 13 to 20 (IBM, Armonk, NY, USA) were used to clean the data according to the survey structure. For quality control purposes, sanitation inspections were carried out by supervisors alongside the data collectors, and their ratings subsequently compared. Five Kato-Katz slides per school (10%) were double-read at the time of sample collection, and a further one slide per school (2%) was retained for further comparison in the central laboratory. The analysis presented here used only data from the original slide readings.

### Data analysis

The aim of this analysis was to quantitatively compare WASH (water practices and sanitation and hygiene facilities) with helminth infection rates. While metrics for the parasitology data—namely, the intensities of infection in either eggs per gram of feces (EPG) or eggs per 10 ml of urine, are readily available, quantifying WASH was less straightforward. The approach adopted is presented below.

#### Calculation of the water score–Inadequate water supplies causing exposure to schistosomes

**Fig 1:**
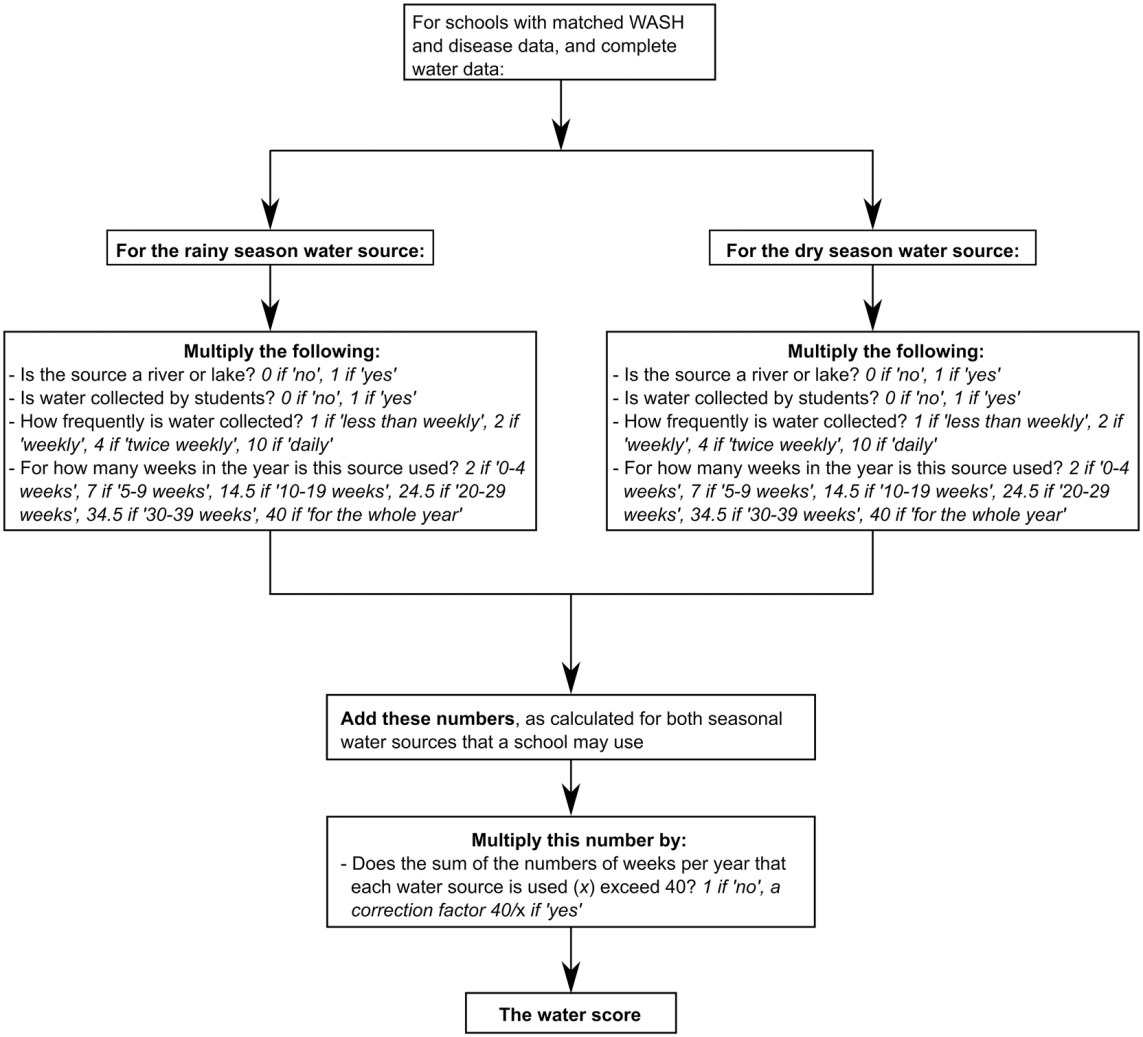
Flow diagram demonstrating the calculation of the water score.

A water score estimated exposure to potentially schistosome-infested water, arising from school-level water collection practices—the calculation of this score is shown in Fig 1. Schools not relying on surface water (i.e., rivers, streams, lakes, or unprotected wells), were assigned a score of zero, as were those in which students did not bring water to the school. In schools whose students brought water from a surface water source, the number of collections per week was multiplied by the number of weeks per year that collections occurred. Where schools relied upon different water sources in the dry and rainy seasons, scores were calculated separately for each season, and the sum was taken. It was assumed that schools were open for a total of 40 weeks per year. Some schools used two sources concurrently, and in some of these schools, the sum of weeks that a school’s two water sources were used for in a year (*x*) exceeded 40. In these cases a correction factor, of 40/*x*, was applied to prevent the total number of school weeks in a year from exceeding 40.

#### Calculation of the sanitation score–Inadequate sanitation causing open defecation and therefore transmission of schistosomes and STHs

**Fig 2:**
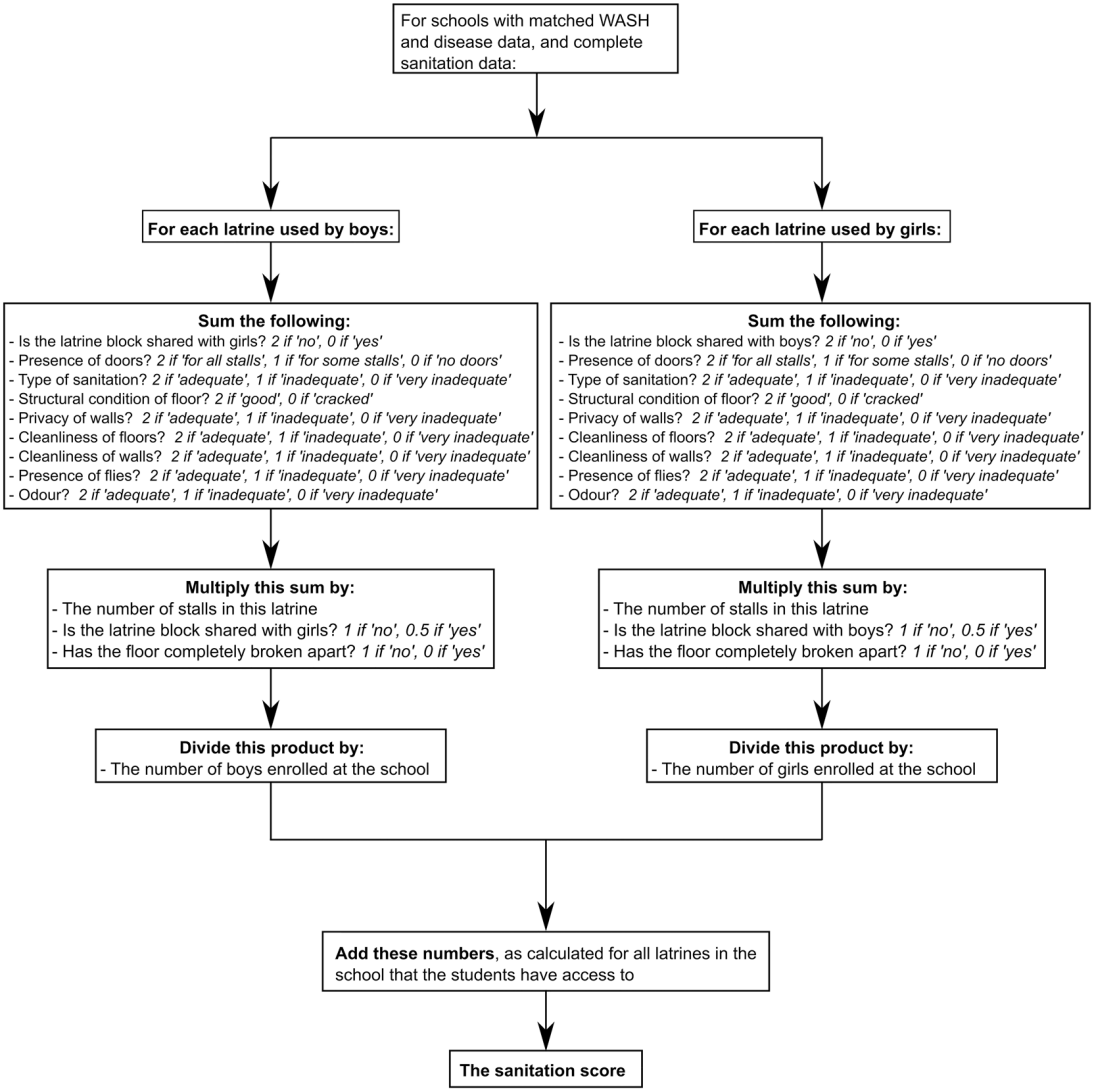
Flow diagram demonstrating the calculation of the sanitation score.

A sanitation score estimated the adequacy of school latrine numbers and conditions. It was calculated as the sum of the ratio of boys’ latrine stalls to boys and the ratio of girls’ latrine stalls to girls, with each latrine’s stalls weighted according to how inoffensive they were on a set of factors. These factors were: being shared with the opposite gender, presence of doors, type of sanitation, structural condition of the floor, privacy of the walls, cleanliness of the floor, cleanliness of the walls, presence of flies, and odor. Each latrine was rated “adequate”, “inadequate”, or “very inadequate”–corresponding to two, one, or zero, respectively, for each aspect. For each latrine, these numbers were summed, then multiplied by the number of stalls (holes) in the latrine. This was then divided by the number of boys or girls in the school. Shared latrines were counted twice, but with their number of stalls halved, on the assumption that boys use half the stalls and girls use the other half. Finally, latrines with collapsed floors were excluded and these numbers were added together for the remaining student latrines. A flow diagram demonstrating this calculation is presented in Fig 2.

Thus the score incorporated both “crowding” and “disgust” elements. Of two schools with equally disgusting latrines, the one with more latrines per student would have a higher score. Of two schools with the same number of latrine stalls, the one with less disgusting latrines would have a higher score. The structure of the score also meant that if a latrine were rated “very inadequate” for all the elements considered, it would contribute nothing to the sanitation score. Regarding type of sanitation, flush toilets, pour flush latrines to septic tanks or latrine pits, and ventilated improved latrines (VIPs) were rated as “adequate”, pit latrines with cement floors and composting latrines were rated as “inadequate”, and pit latrines without cement floors, hanging latrines, and pour flush latrines to other locations, were rated as “very inadequate”. More details of the exact definitions of “adequate”, “inadequate”, and “very inadequate” for the sanitation elements may be seen in the results.

#### Calculation of the hygiene score–Inadequate handwashing facilities promoting the transmission of STHs

**Fig 3:**
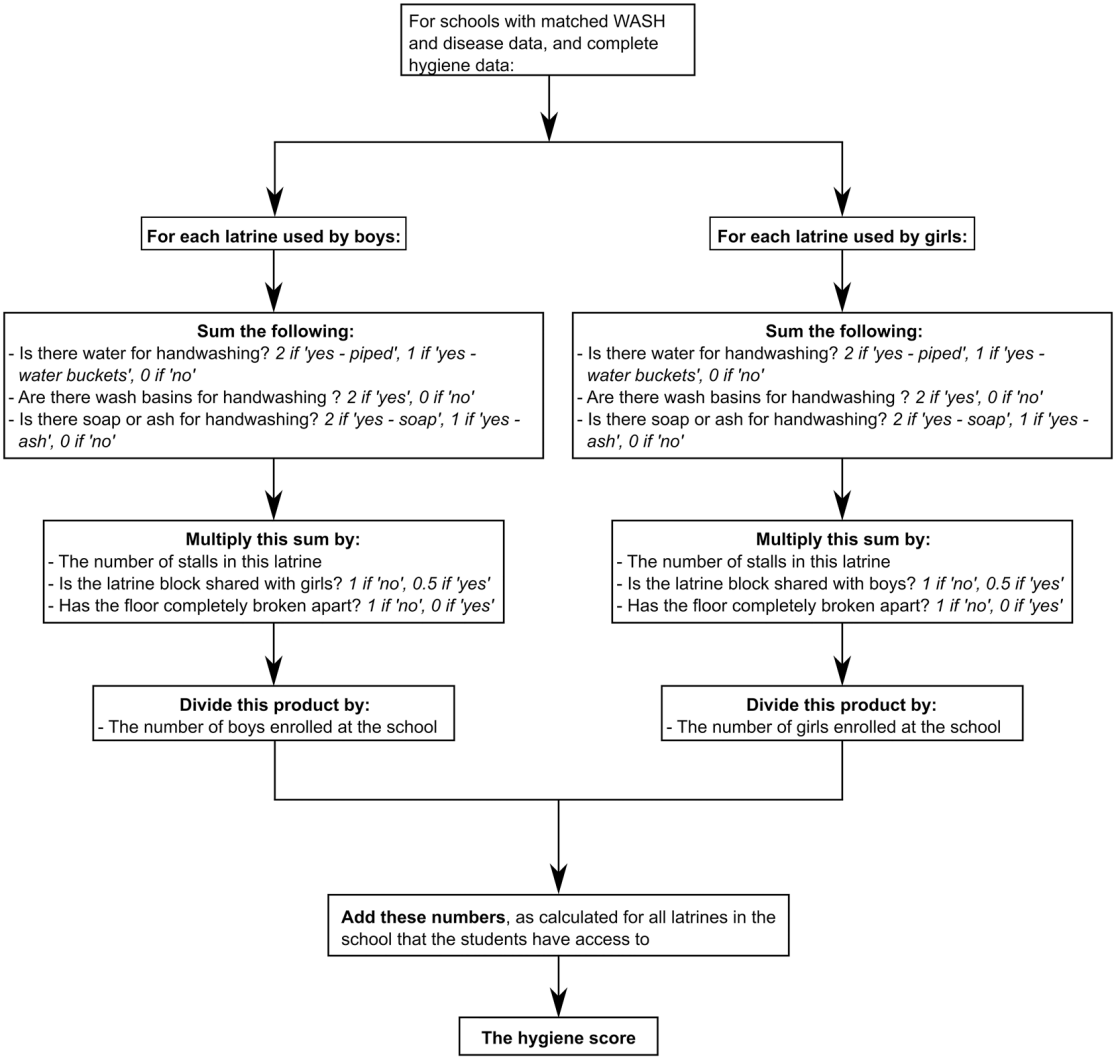
Flow diagram demonstrating the calculation of the hygiene score.

A hygiene score was constructed in a similar manner to the sanitation score above, except that rather than rating latrine blocks according to the disgust level, this score rated them according to the provision of water and soap, and the presence of handwashing basins. A flow diagram demonstrating this calculation is presented in Fig 3.

#### Calculation of the combined water and sanitation, and sanitation and hygiene scores

Possible associations between the infection intensities, and combinations of water, sanitation, and hygiene were also investigated. After excluding the top 5% of the sanitation and the hygiene scores, the water, sanitation, and hygiene scores were normalized by dividing by their respective new maxima. This exclusion of the top 5% of schools mitigated any impact of a few very high sanitation and hygiene scores on their normalization. Such high scores resulted from errors in the collection and input of data, in particular, those pertaining to the numbers of students and of latrines. The lowest scores, on the other hand, were not excluded. This is because the minimal score of zero was more plausible (reflecting either no latrines or no hygiene provisions), and because only the maximum scores were used in this normalization. A new water score was defined as this normalized water score, subtracted from one (so that higher values of the new water score reflected less potential exposure to schistosomes during water collection). The new water and sanitation, and the sanitation and hygiene scores were then added to give combined scores.

#### Linking of WASH and parasitology data

School-level arithmetic means were calculated for the infection intensities (in EPG) of the STHs and *S*. *mansoni*. Intensities, rather than prevalences, were used since they are more reflective of both cumulative exposure and morbidity, and since small changes in prevalence may be accompanied by large changes in intensity [19]. Given the extremely low *S*. *haematobium* prevalence (0.2% in all children tested), it was not compared with WASH. The parasitology and WASH data were matched and school names in the two databases were used to verify that the databases had been matched correctly. Schools with different names in the two databases were excluded from the analyses. Schools with missing enrolment data, and those in which fewer than 25 students were tested, were also excluded. A flow diagram showing these exclusions in more detail is provided in S1 Fig.

#### Statistical tests

In all analyses, schools with 0% prevalence of the parasite in question were excluded, on the assumption that the complete absence of infection most likely results from non-WASH factors such as temperature, precipitation, soil type, and proximity to water bodies harboring intermediate host snails [1,5,12,13,20,21]. Excellent school WASH facilities and practices might prevent some infections with these parasites. However, it is likely that even with the best possible school WASH facilities and practices, children might not *always* practice perfect WASH-related behaviors, and might be exposed to the parasites away from school. To investigate this further, the Mann-Whitney *U* test [22], a non-parametric test that compares ranks in a dependent variable, between two groups defined by a nominal independent variable, was used. Mann-Whitney *U* tests compared the water, sanitation, and hygiene scores between the schools with zero, and non-zero, prevalences of infection with each parasite. The results of these tests are summarized in S1 Table of the supplementary data.

Kendall’s *τ*_*b*_ [23,24], another non-parametric statistical test that compares ranks between two variables and corrects for tied values, was used to test the non-zero prevalence schools’ WASH scores against their arithmetic mean intensities (in EPG). This statistic has been used in previous studies analyzing different risk factors for helminth infection [25,26]. Each WASH score was compared with the parasites whose life cycles provide a rationale for an association between the WASH score and the parasitic infection intensity; the water score (estimating exposure to potentially schistosome-infested water) was compared with mean intensity of *S*. *mansoni*, the sanitation score (estimating adequacy and thus a proxy for use of sanitation) with the mean intensities of *S*. *mansoni* and the STHs, and the hygiene score (estimating handwashing provisions) with those of the STHs. Similarly, the combined water and sanitation score was tested against the mean intensity of *S*. *mansoni*, and the combined sanitation and hygiene score was tested against the mean intensities of *A*. *lumbricoides*, *T*. *trichiura*, and hookworm. *P* values lower than 0.05 were considered statistically significant.

Mann-Whitney *U* tests, which have also previously been employed in the assessment of risk factors for helminth infection [27–29], were used to compare arithmetic mean infection intensities of *S*. *mansoni* and the STHs between schools with and without evidence of open defecation in the compound. Once more, *P* values lower than 0.05 were considered statistically significant.

Age was not accounted for in any of the analyses, since the age band was narrow (over 98% of schools had a mean age between 11 and 13, when rounded to the nearest year). These ages are not generally characterized by rapid increases in worm burdens [30], and while hookworm and schistosome infection intensities in a given community frequently rise with age [1,3], this trend does not apply across communities. Indeed, no clear trends are apparent in the plots of school-level mean intensity of infection against school-level mean age of those tested (shown in S2 Fig). Similarly, data were not stratified by gender since having excluded schools with zero prevalence, the arithmetic mean of differences between the genders’ (boys’ minus girls’) school-level arithmetic mean infection intensities were 8.6, 9.2, -27.6, and -0.4 for *S*. *mansoni*, hookworm, *A*. *lumbricoides*, and *T*. *trichiura*, respectively, while the standard deviations on these values were much higher (48.3, 99.4, 798.6, and 66.5, respectively).

All analyses were conducted in R version 0.98.1091 (R Foundation for Statistical Computing, Vienna, Austria), and the Hmisc, plotrix and plyr packages were used.

## Results

### Parasitology summary, and number of schools with complete data

Kato-Katz data were available for 116,042 schoolchildren in 2,471 schools. Urine samples from 99,726 children in 2,007 schools were collected and tested for hematuria, with Hemastix. Filtration and microscopy was carried out on the 70,669 urine samples (from 1,697 schools) that were positive for hematuria.

Excluding schools with samples from fewer than 25 students left 115,052 students in 2,342 schools who provided stool samples, and 99,137 students in 1,887 schools who provided urine samples. In these students, the prevalences of *S*. *mansoni*, *S*. *haematobium*, *A*. *lumbricoides*, *T*. *trichiura*, and hookworm were 3.5%, 0.2%, 13.3%, 7.8%, and 7.4%, respectively. At least one case of each of those helminths was found in 24.4%, 3.1%, 75.4%, 51.7%, and 52.0% of these schools, respectively. As a result of the scarcity of *S*. *haematobium* cases (176 children in 58 schools), we decided not to compare this parasitic infection with school WASH.

WASH data were collected in 2,323 schools. However, only 1,876 schools’ WASH and enrolment data (needed for the calculation of the sanitation and hygiene scores) could be reliably matched—that is, schools with the same identification number in the two databases had similar names in the two databases. Only 1,645 of those 1,876 schools could be matched to schools in the Kato-Katz database. Mann-Whitney *U* tests comparing WASH scores between the 1,645 included schools, and the 231 schools without parasitology data did reveal some statistically significant differences. Similarly, Mann-Whitney *U* tests comparing mean parasite infection intensities between the 1,645 included schools, and the 530 schools without WASH scores, also revealed some statistically significant differences. The results of these tests are shown in S1 Table of the supplementary data.

**Fig 4:**
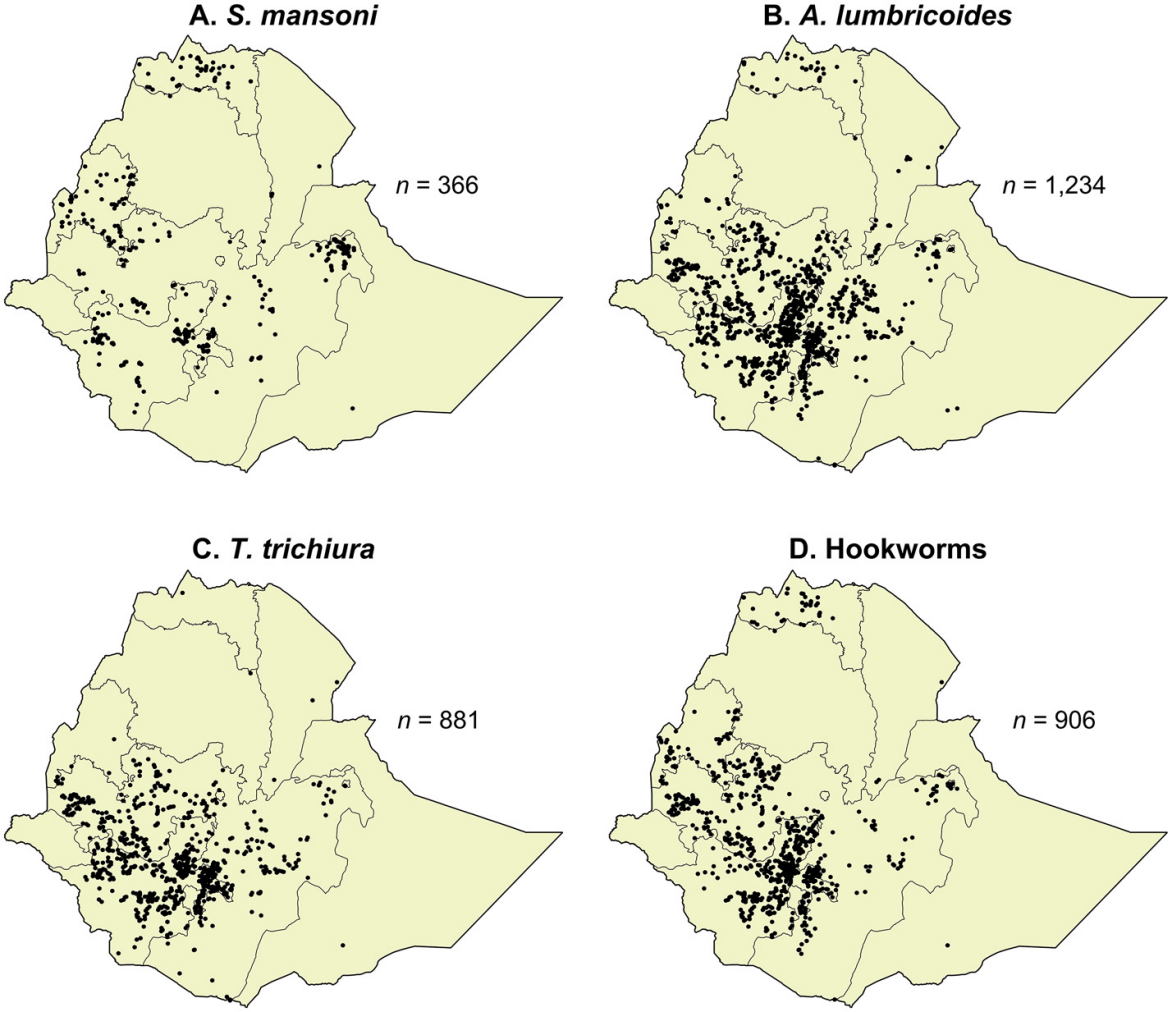
Locations within Ethiopia of the schools with WASH data and non-zero mean intensity of infection with A: *S*. *mansoni*, B: *A*. *lumbricoides*, C: *T*. *trichiura*, and D: hookworm. The number of schools (*n*) is also provided for each map. Note that some of the schools in these maps were not used in the analyses because their water, sanitation, or hygiene data were incomplete.

Of the 1,645 schools with matched WASH, enrolment, and parasitological data, *S*. *mansoni*, *A*. *lumbricoides*, *T*. *trichiura*, and hookworms were present in 366, 1,234, 881, and 906 schools, respectively. The locations of schools with WASH data, and non-zero prevalences of each parasite, are shown in Fig 4. Mann-Whitney *U* tests were used to compare WASH scores between the included schools, and those excluded for having zero prevalence of infection with each parasite. These tests did reveal some statistically significant differences, and the results are shown in S2 Table of the supplementary data.

### WASH summary

Water, sanitation, and hygiene characteristics of the 1,645 schools with matched WASH, enrolment, and Kato-Katz data are summarized below.

#### Water

On-site year-round sources were available in 748 (45.5%) schools, while 61 (3.7%) had rainy-season only on-site sources, and 836 (50.8%) had no on-site source. The breakdown of schools’ primary water sources in the rainy and dry seasons is presented in Table 1.

**Table 1:** Schools' primary water sources in the rainy and dry seasons.

Water source	Rainy season^a^		Dry season^b^	
	Number of schools	Percentage	Number of schools	Percentage
**Surface water** (from rivers, ponds or lakes)	469	28.5%	496	30.2%
**Borehole, tubewell, or protected dug well**	197	12.0%	194	11.8%
**Standpipe**	450	27.4%	451	27.4%
**Rainwater**	77	4.7%	41	2.5%
**Protected spring**	109	6.6%	115	7.0%
**Unprotected dug well**	36	2.2%	37	2.2%
**Piped water**	287	17.4%	290	17.6%
**Other**	4	0.2%	8	0.5%

^a^ Data missing for 16 schools, 1.0%

^b^ Data missing for 13 schools, 0.8%

A total of 520 schools (31.6%, data missing for 14 schools) made use of surface water. There were 845 schools (51.4%) in which, as a result of the absence of an on-site water source for at least some of the year, children were required to contribute to the school’s water supplies by bringing water from elsewhere. Water was used for bathing in 389 schools (23.6%). The water score could not be calculated for 171 schools, due to missing data. For the remaining schools, the mean water score was 54.0 (standard deviation, SD = 113.5, range: 0 to 400). School-level water collection practices did not cause any exposure to schistosomes in 1,027 schools (69.7%), reflected by a water score of zero in these schools.

#### Sanitation and hygiene

These 1,645 schools had a total of 2,967 latrine blocks for students—corresponding to a mean of 1.8 student latrine blocks per school (SD = 0.7, user data missing for 169 latrines). Separate latrine blocks for boys and girls were present in 1,127 schools (68.5%). On average, there were 0.0081 boys’ latrine stalls per boy, 0.0065 girls’ latrine stalls per girl, and 0.0019 shared latrine stalls per child, and the SDs on these figures were relatively high (0.011, 0.0096, and 0.0049, respectively).

These 2,967 latrines were most commonly pit latrines with cement slabs (1,886 latrines, 63.6%). Other forms of latrine included pit latrines without cement slabs (722 latrines, 24.3%), VIP latrines (166 latrines, 5.6%), composting toilets (six latrines, 0.2%), pour flush latrines to pits (six latrines, 0.2%), pour flush latrines to elsewhere (four latrines, 0.1%), and hanging latrines (eight latrines, 0.3%). Data on the kind of sanitation were missing for a further 169 latrines (5.7%). Summary data on latrine conditions are presented in Table 2. Of note, many of the sanitation and hygiene conditions were highly associated by *χ*^2^ (data not presented).

**Table 2:** Summary of aspects of latrine conditions used in the calculation of the sanitation and hygiene scores.

Indicator	Adequate			Inadequate			Very inadequate			Data missing	
	Definition	Number of latrines	Percentage	Definition	Number of latrines	Percentage	Definition	Number of latrines	Percentage	Number of schools	Percentage
**Type of sanitation**	Flush toilets, pour flush latrines to septic tanks or latrine pits, and ventilated improved pit latrines	172	5.8%	Pit latrines with cement floors and composting latrines	1892	63.8%	Pit latrines without cement floors, hanging latrines, and pour flush latrines to “other locations”	734	24.7%	169	5.7%
**Presence of doors**	Doors for all stalls	1,576	53.1%	Doors for some, but not all, stalls	383	12.9%	No doors at all	391	13.2%	617	20.8%
**Sharing with opposite gender**	Is not shared with opposite gender	2,514	84.7%	-	-	-	Is shared with opposite gender	453	15.3%	0	0
**Structural condition of floor**^a^	Floor shows no signs of damage	1,927	65.0%	Floor is cracked but in place	698	23.5%	Floor has broken into separate pieces and fallen into the pit	118	4.0%	224	7.5%
**Privacy of walls**	Fully private walls	1,914	64.5%	Walls with holes	671	22.6%	No walls	180	6.1%	202	6.8%
**Cleanliness of floors**	Clean (no feces, urine, dirt, or refuse)	528	17.8%	Unclean (some feces, urine, dirt, or refuse)	1,802	60.7%	Very unclean (major presence of feces, urine, dirt, or refuse)	416	14.0%	221	7.4%
**Cleanliness of walls**	Clean (no feces, urine, or dirt)	1,056	35.6%	Unclean (some feces, urine, or dirt)	1,492	50.3%	Very unclean (major presence of feces, urine, or dirt)	209	7.0%	210	7.1%
**Flies**	No flies	515	17.4%	Some flies (less than about 20 per stall)	1,851	62.4%	Many flies (more than about 20 per stall)	389	13.1%	212	7.1%
**Odor**	No odor	361	12.2%	Slightly intolerable odor	1,879	63.3%	Highly intolerable odor	507	17.1%	220	7.4%
**Water for handwashing**	Piped water for handwashing at the latrine	97	3.3%	Bucket water for handwashing at the latrine	205	6.9%	No water for handwashing at the latrine	2,450	82.6%	215	7.2%
**Washbasins for handwashing**	Washbasins for handwashing at the latrine	130	4.4%	-	-	-	No washbasins for handwashing at the latrine	2,584	87.1%	253	8.5%
**Soap or ash for handwashing**	Soap for handwashing at the latrine	46	1.6%	Ash for handwashing at the latrine	29	1.0%	Neither soap nor ash for handwashing at the latrine	2,655	89.5%	237	8.0%

^a^Note that in the calculation of the sanitation score, latrines with floors showing no signs of damage were assigned two, and those with floors that had cracked but in place were assigned zero, while those with floors that had broken into separate pieces and fallen into the pit were excluded from the calculation of the score.

The mean school sanitation score was 0.23 (SD = 0.37, range: 0 to 10.1, data missing for 481 schools, 29.2%). The sanitation score was zero for 93 schools (5.7%). The school hygiene score had a mean of 0.0060 (SD = 0.027, range: 0 to 0.48, data missing for 332 schools, 20.1%), and it was zero for 1,156 schools (70.3%). Evidence of open defecation was observed in 661 schools (40.2%, data missing for 43 schools, 2.6%).

For quality control, the supervisors independently inspected school sanitation and hygiene alongside the enumerators in 12 schools. For the indicators used in the calculation of the sanitation and hygiene scores (i.e., those in Table 2), ratings were in agreement in 61.8% of cases. A further 34.5% were different but belonged to the adjacent class on the “adequate”, “inadequate”, and “very inadequate” scale, while 3.8% were two classes apart.

### Water and *S*. *mansoni*

**Fig 5:**
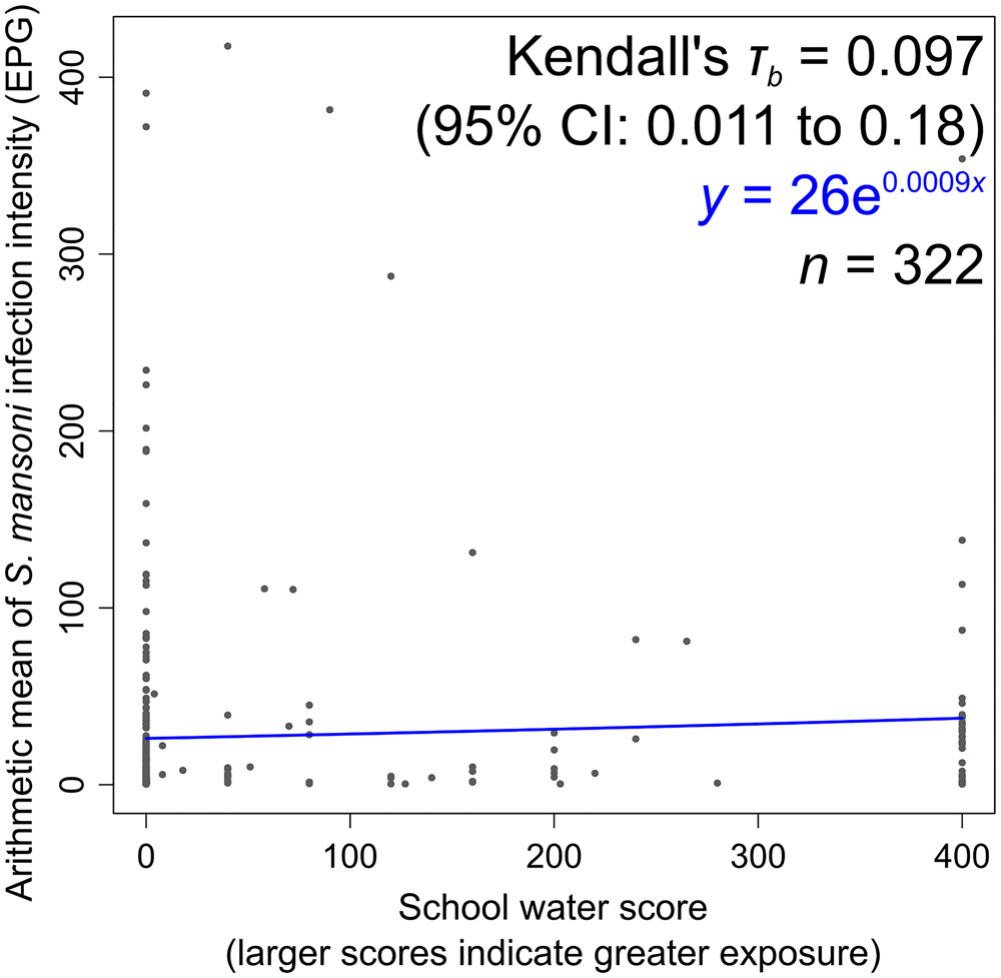
School water scores and arithmetic mean intensities of *S*. *mansoni* infection. Kendall’s *τ*_*b*_ statistics, the equation of the least-squares line of best fit, and the number of included schools, are presented in the upper-right corner.

Of the 366 schools with WASH data and non-zero *S*. *mansoni* prevalences, 322 had complete data on school-level water collection practices causing potential exposure to schistosomes. Schools with higher water scores (i.e. more frequent collection of water from potentially schistosome-infested sources) had significantly higher arithmetic mean *S*. *mansoni* infection intensities (Kendall’s *τ*_*b*_ = 0.097, 95% CI: 0.011 to 0.18). Their water scores are plotted against their *S*. *mansoni* infection intensities in Fig 5.

### Sanitation, *S*. *mansoni*, and the STHs

**Fig 6:**
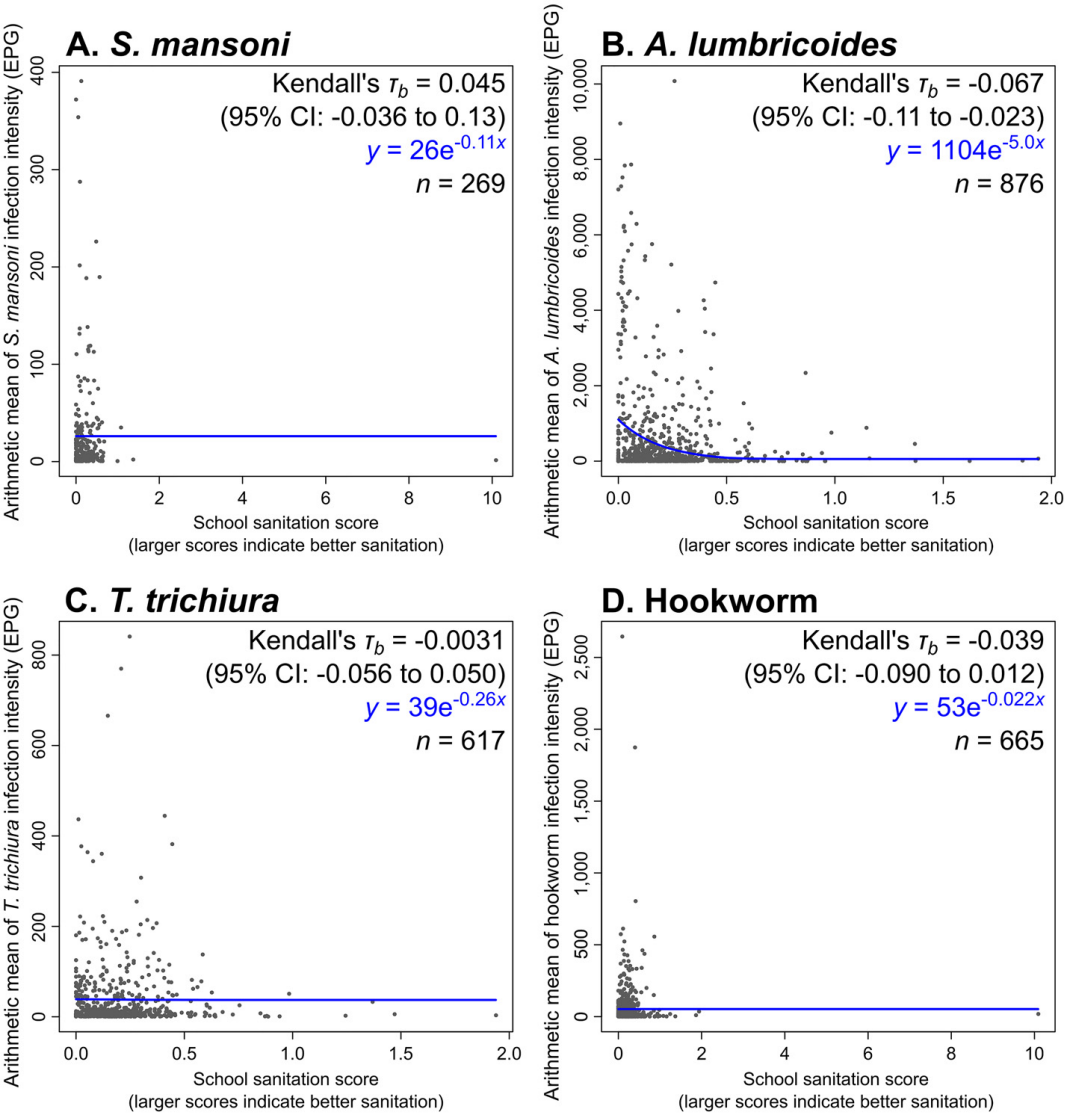
School sanitation scores against their arithmetic mean infection intensities for A. *S*. *mansoni*, B. *A*. *lumbricoides*, C. *T*. *trichiura*, and D. hookworm. The Kendall’s *τ*_*b*_ statistics, equation of the least-squares line of best fit, and sample size are presented in the upper-right corner of each graph.

There were 366, 1,234, 881, and 906 schools with WASH data and at least one case of *S*. *mansoni*, *A*. *lumbricoides*, *T*. *trichiura*, and hookworms, respectively. Considering schools with complete sanitation data and at least one case of each parasite, these figures were 269, 876, 617, and 665 schools, respectively. Higher sanitation scores (indicating the presence of more latrine stalls per student, and in better condition), were associated with significantly lower intensities of *A*. *lumbricoides* infection (Kendall’s *τ*_*b*_ = -0.067, 95% CI: -0.11 to -0.023). No statistically significant differences were observed for *S*. *mansoni* (Kendall’s *τ*_*b*_ = 0.045, 95% CI: -0.036 to 0.13), *T*. *trichiura* (Kendall’s *τ*_*b*_ = -0.0031, 95% CI: -0.056 to 0.050), or hookworm (Kendall’s *τ*_*b*_ = -0.039, 95% CI: -0.090 to 0.012), although in the case of hookworm, the difference was borderline statistically significant (*P* = 0.067). These schools’ sanitation scores are plotted against their average infection intensities in Fig 6.

### Hygiene (handwashing following defecation) and the STHs

**Fig 7:**
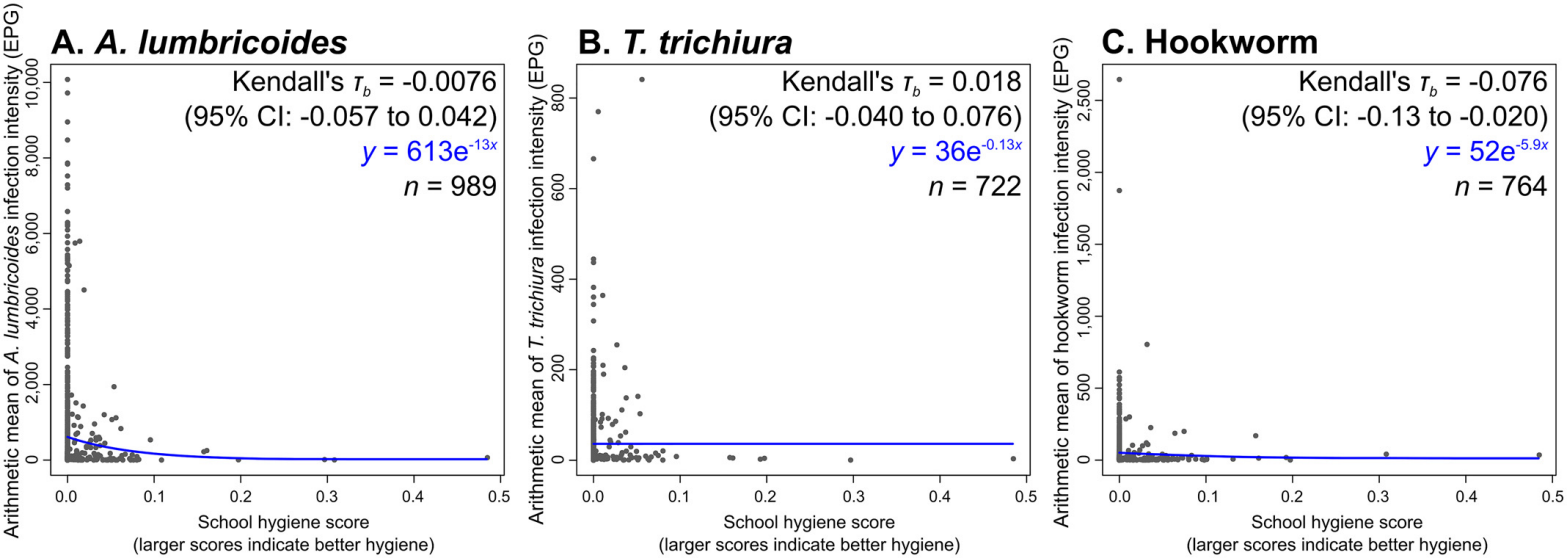
School hygiene scores against their arithmetic mean infection intensities for A. *A*. *lumbricoides*, B. *T*. *trichiura*, and C. hookworm. The Kendall’s *τ*_*b*_ statistics, least-squares best fit line equation, and sample size are presented in the upper-right corner of each graph.

There were 1,234, 881, and 906 schools with WASH data and some *A*. *lumbricoides*, *T*. *trichiura*, and hookworms, respectively. Considering schools with complete hygiene data and at least one case of each helminth, these figures were 989, 722, and 764 schools, respectively. Schools with higher hygiene scores (i.e. those with better availability of latrines with soap or ash, basins, and water for handwashing) had significantly less hookworm (Kendall’s *τ*_*b*_ = -0.076, 95% CI: -0.13 to -0.020). While the mean *A*. *lumbricoides* and *T*. *trichiura* infection intensities were lower for schools with higher hygiene scores (by Kendall’s *τ*_*b*_ test and the lines of best fit), the difference was not statistically significant (Kendall’s *τ*_*b*_ = -0.0076, 95% CI: -0.057 to 0.042 and Kendall’s *τ*_*b*_ = 0.018, 95% CI: -0.040 to 0.076, respectively). These schools’ hygiene scores are plotted against their average infection intensities in Fig 7.

### Combined water and sanitation, and sanitation and hygiene scores

After excluding schools missing water or sanitation data, with 0% *S*. *mansoni* prevalence, and those with the top 5% of water and sanitation scores, 242 schools remained. The combined water and sanitation score was not significantly associated with the arithmetic mean intensity of *S*. *mansoni* infection (Kendall’s *τ*_*b*_ = 0.034, 95% CI: -0.052 to 0.12). A graph of arithmetic mean *S*. *mansoni* infection intensity against the combined water and sanitation score, along with the Kendall’s *τ*_*b*_ statistics, is shown in S3 Fig.

Having excluded schools missing sanitation or hygiene data, with 0% prevalence or each parasite, and those with the top 5% of sanitation and hygiene scores, 788, 556, and 586 schools remained for *A*. *lumbricoides*, *T*. *trichiura*, and hookworm, respectively. Higher combined sanitation and hygiene scores were associated with significantly lower mean infection intensities of *A*. *lumbricoides* (Kendall’s *τ*_*b*_ = 0.047, 95% CI: -0.094 to -0.00063), but not *T*. *trichiura* (Kendall’s *τ*_*b*_ = 0.021, 95% CI: -0.035 to 0.077), or hookworm (Kendall’s *τ*_*b*_ = -0.049, 95% CI: -0.10 to 0.0055). Graphs of the arithmetic mean STH infection intensities against combined sanitation and hygiene scores are provided in S4 Fig.

### Evidence of open defecation, *S*. *mansoni*, and the STHs

The results of the Mann-Whitney *U* tests, comparing helminth infection intensities with evidence of open defecation within the school compound, are shown in Table 3. Evidence of open defecation was associated with significantly more *T*. *trichiura* (*P* < 0.05), but with significantly less *A*. *lumbricoides* (*P* < 0.001), and with no statistically significant differences in *S*. *mansoni* or hookworm (*P* > 0.05 for both parasites).

**Table 3:** Mann-Whitney *U* tests comparing infection rates in schools with and without evidence of open defecation.

Parasite	Number of schools with some infection, and evidence of open defecation	Median of arithmetic mean infection intensity in schools with some infection, and evidence of open defecation	Number of schools with some infection, but no evidence of open defecation	Median of arithmetic mean infection intensity in schools with some infection, but no evidence of open defecation	Mann-Whitney *U*	*P* value
*S*. *mansoni*	114	7.4	140	6.5	7786	> 0.05
*A*. *lumbricoides*	347	47.5	516	82.8	75,511	< 0.0001
*T*. *trichiura*	210	11.5	398	8.8	45,960	< 0.05
Hookworm	230	12.5	423	12.2	50,727	> 0.05

## Discussion

The collection and analysis of data on a national scale has enabled a statistically powerful comparison of school WASH with school-level arithmetic mean intensities of infection with *S*. *mansoni* and the STHs. Even with *S*. *mansoni*, a parasite known to occur focally in space, we were able to include 15,874 students in 322 schools in our water analysis. The results of this analysis are suggestive of impacts of WASH on the parasites, and we hope this evidence will help to guide future decision-making on the integration of different school health programs.

The statistically significant association between the water score and *S*. *mansoni* infection intensity suggests that compelling children to bring water to school from open water bodies may cause measurably (if only slightly) higher intensities of infection. It is understood that water collection usually causes a relatively small fraction of the total exposure to schistosomes, while other activities such as recreational swimming, bathing, and laundry are often more important [5]. Indeed, an alternative explanation of this finding is that the proximity of schools to open water bodies might drive the use of the latter both as a school water source, and for recreational swimming and bathing. Intervention studies, combining school safe water supply provision with the monitoring of schistosome infection rates, would reveal the relative importances of these different possible causes of association.

Concerning sanitation, a recent study carried out in Kenya found that schools with more latrines per pupil and with newer latrines had significantly higher latrine usage rates (*P* < 0.01 when comparing adjusted incidence rate ratios) [31]. Increased use of sanitation (which was unfortunately unfeasible to measure directly in this study) should reduce open defecation in and around the school compound, and might therefore be expected to reduce transmission of schistosomes and the STHs. On the other hand, it is possible that inadequately built or maintained latrines may exacerbate STH transmission, by concentrating defecation in one place, and bringing students into that place. This concern has previously been raised for hookworm [7], but it could also apply to *A*. *lumbricoides* and potentially to *T*. *trichiura*, particularly in simple pit latrines without cement floors [32,33].

We found that schools with better sanitation did have statistically significantly lower *A*. *lumbricoides*, and borderline significantly lower hookworm, infection intensities. Anderson *et al*. (2013) have shown that children produce a smaller proportion of hookworm eggs than of *A*. *lumbricoides* eggs [34]. By extension, perhaps exposure around the school compound is more important in the transmission of *A*. *lumbricoides*, while a greater proportion of hookworm infections occur elsewhere, as a result of larvae deposited by infected adults, rather than by children. An alternative explanation is that delays in the preparation and reading of Kato-Katz slides, may have led to a lower sensitivity for hookworm infections [17,18]. This might have hindered our ability to detect an association with sanitation conditions.

Although *T*. *trichiura* and *A*. *lumbricoides* follow very similar life cycles, and we found a significant association between sanitation and *A*. *lumbricoides*, we found none between sanitation and *T*. *trichiura*. This may be due to differences between the parameters of these parasites’ transmission. *T*. *trichiura* has been described as “intrinsically more resistant to control than either hookworm or *A*. *lumbricoides*” [33] on account of its higher basic reproduction ratio (*R*_0_). *A*. *lumbricoides* eggs survive for longer than do those of *T*. *trichiura*, and the latter also have lower lethal and optimal embryonation temperatures [33,35]. Perhaps therefore temperature is a more important limit on *T*. *trichiura* transmission, while that of *A*. *lumbricoides* is more amenable to control through containment of the eggs. Another possibility is that the slightly lower sensitivity of the Kato-Katz technique for *T*. *trichiura* infections [36,37] may have hampered our ability to detect any association with sanitation conditions—particularly since it was feasible only to examine one slide from one stool specimen per participant in this survey.

We found no statistically significant association between sanitation score and mean intensity of *S*. *mansoni* infection. This is likely due to the fact that for transmission to occur, eggs in the definitive host’s feces must enter freshwater containing intermediate host snails [3]. Inadequate sanitation leading to open defecation in and around the school compound does not imply that feces will enter freshwater bodies.

The finding that schools with evidence of open defecation had significantly lower *A*. *lumbricoides*, but significantly higher *T*. *trichiura* infection intensities, appears to contradict the associations between these parasites and the sanitation score. Open defecation at school likely takes place in many different sites, as found in an Egyptian village [38]. It is therefore possible that data collectors may have missed feces in many schools, or they may have found evidence of open defecation in schools that until recently had been very clean. Once more, the lack of a statistically significant association between this indicator and *S*. *mansoni* infection intensities was not surprising, given its life cycle, which is unlikely to be completed within the school compound.

The statistically significantly lower hookworm infection intensities in schools with better hygiene, is difficult to explain, particularly given the lack of significant associations between hygiene and *A*. *lumbricoides* or *T*. *trichiura* infection intensities. Although *A*. *duodenale* infection can occur orally, hookworm infections generally result from dermal contact with the larvae, while *A*. *lumbricoides* and *T*. *trichiura* infections are caused only by ingestion of the eggs. Handwashing might therefore be expected to have the most pronounced impacts on the transmission of *A*. *lumbricoides* and *T*. *trichiura*, rather than that of hookworm. This analysis was affected by the very large proportion of schools with no hygiene facilities (70.3% had hygiene scores of zero), as well as the very low infection rates in the few schools with good hygiene. It may be an example of a ‘false discovery’–that is, a statistically significant trend in the data due to chance rather than to any relationship between handwashing in school and hookworm infection intensity [39].

This study suffered several limitations. Transmission, particularly of the STHs, likely occurs around the home as well as the school, and in other areas. However, it was unfortunately unfeasible to assess household WASH in this project. Furthermore, it is possible that the various sanitation condition factors (privacy, doors, intolerable odors, flies, damaged floors, and unclean walls and floors) are not equally important in the determination of sanitation disgustingness and use. However, we could find no studies on the relative importance of these factors in determining how likely students are to use latrines. Also, although rating sanitation conditions on the semi-quantitative scale of “adequate”, “inadequate”, and “very inadequate” is necessarily subjective, the 61.8% exact agreement between data collectors and supervisors was slightly lower than in a similar previous project in southern Ethiopia, where agreement rates were around 70%. Because of the scale of this project, supervision was necessarily less close, and it was not feasible to include a school visit as part of the training. These factors may have contributed to the difference.

Our analyses have excluded many schools: some had incomplete WASH data or could not be found in the parasitology database, and others had zero prevalence for the parasitic infection in question. Schools excluded because they had incomplete WASH data exhibited some statistically significant differences in infection intensities. Similarly, those with no parasitology data had some significantly different WASH scores to the included schools. There were also some significant differences in WASH scores between the schools with and without zero prevalence of each parasitic infection. However, the magnitudes of the differences between included and excluded schools were small. Furthermore, for some parasite-WASH combinations, the schools with zero prevalence had lower WASH scores, while for others, the schools with zero prevalence had higher WASH scores. The fact that these tests revealed no strong or coherent trends (such as consistent and large differences between the median scores), suggests that perhaps geographical variation in both parasitic infection rates and data collectors, and large sample sizes, may have given rise to these statistically significant differences.

We found statistically significant associations between school water collection practices and *S*. *mansoni*, school sanitation and *A*. *lumbricoides*, and school hygiene and hookworm. However, the associations were somewhat moderate, as reflected by the small Kendall’s *τ*_*b*_ values. Children also spend time (and become infected with these parasites) outside of school. This may partly account for these low values. Furthermore, the data are observational. The results consequently represent the strengths of current associations, rather than the potential impacts of WASH on the parasites. On the other hand, there are many local social and environmental factors that modulate (and thus perhaps weaken) the impact of WASH on the parasites’ transmission.

WASH is a key component of school health and nutrition interventions, and some of its benefits are well known [4]. This analysis suggests that certain aspects of WASH may help to prevent the transmission of *S*. *mansoni* around, and STHs within, schools—lending support for its inclusion in school health and nutrition programs. Future studies should explore whether these associations hold in different countries, and in the contexts of WASH interventions and of reinfection following PC. Investigation of programmatic questions, such as the cost-effectiveness of large-scale WASH programs *versus* PC in the control of STHs, is also needed to ensure that these programs achieve maximal health benefits.

## Supporting Information

S1 Fig Flow diagram showing the manipulation of the various datasets. (JPG)

S2 Fig Arithmetic mean intensity of infection against arithmetic mean of the age of those tested for A. *S*. *mansoni*, B. *A*. *lumbricoides*, C. *T*. *trichiura*, and D. hookworm, in schools with non-zero prevalences of each parasite. The lines of best fit are shown in blue. Their equations, the coefficients of determination (*R*^2^) and the number of schools (*n*) are presented in the upper-right corner of each graph.
(JPG)

S3 Fig School water and sanitation combined scores against their arithmetic mean *S*. *mansoni* infection intensities. Kendall’s *τ*_*b*_ statistics, the equation of the least-squares line of best fit, and the number of included schools, are presented in the upper-right corner.
(JPG)

S4 Fig School sanitation and hygiene combined scores against their arithmetic mean infection intensities for A. *A*. *lumbricoides*, B. *T*. *trichiura*, and C. hookworm. The Kendall’s *τ*_*b*_ statistics, least-squares best fit line equation, and sample size are presented in the upper-right corner of each graph.
(JPG)

S1 Table Comparisons of infection intensities and WASH scores between the schools included in the analysis, and those that were excluded since they could not be matched to the other database. (XLSX)

S2 Table Comparisons of WASH scores between schools with zero and non-zero prevalences of each parasite. (XLSX)
