# Supplementary figures and images for: School Water, Sanitation, and Hygiene, Soil-Transmitted Helminths, and Schistosomes: National Mapping in Ethiopia
Source: PLoS Negl Trop Dis. 2016 Mar 8;10(3):e0004515. doi: 10.1371/journal.pntd.0004515 (PMC4783033; doi:10.1371/journal.pntd.0004515)

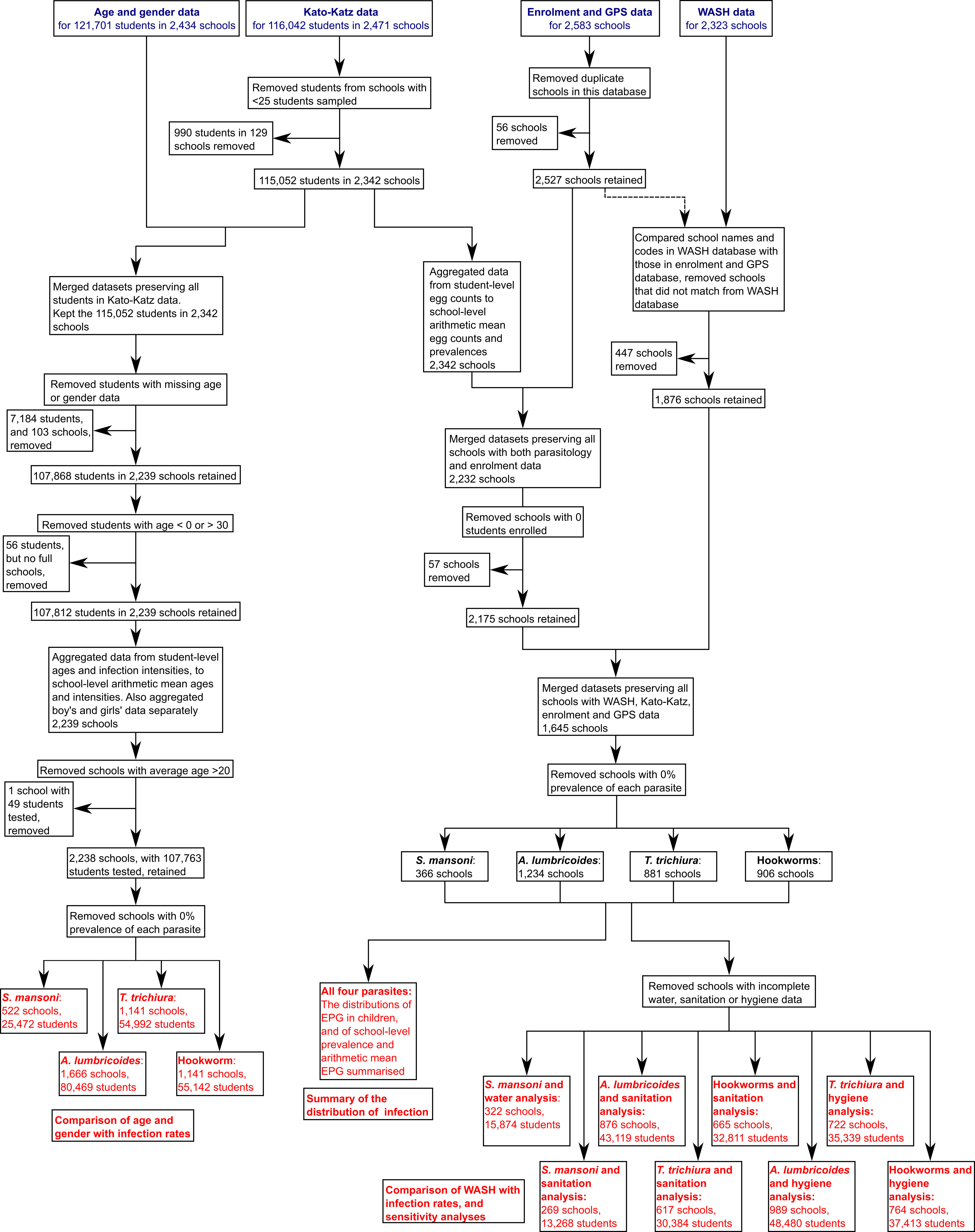

Supplement: S1 Fig — (JPG) [file pntd.0004515.s001.jpg]

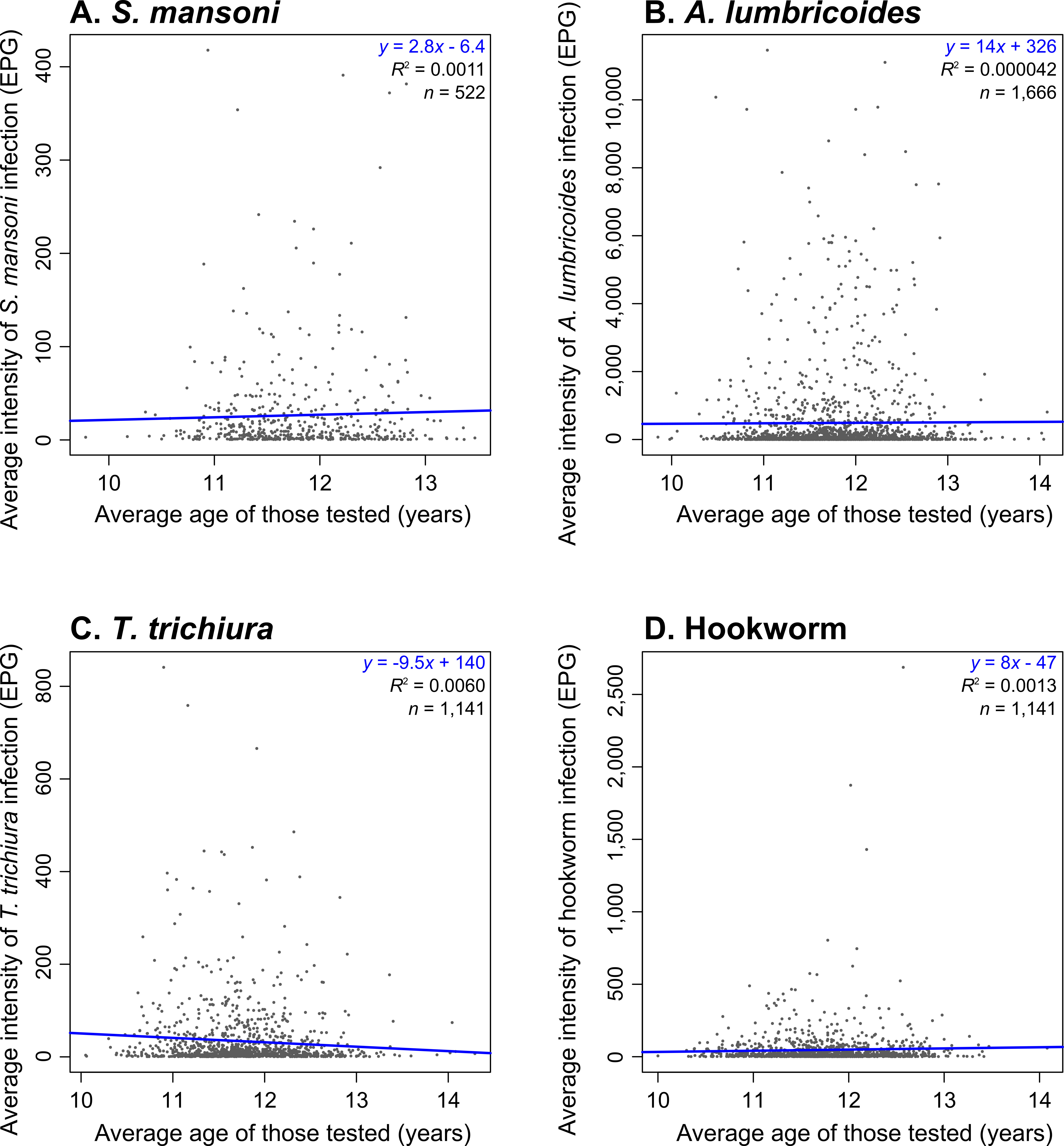

Supplement: S2 Fig — The lines of best fit are shown in blue. Their equations, the coefficients of determination (R2) and the number of schools (n) are presented in the upper-right corner of each graph. (JPG) [file pntd.0004515.s002.jpg]

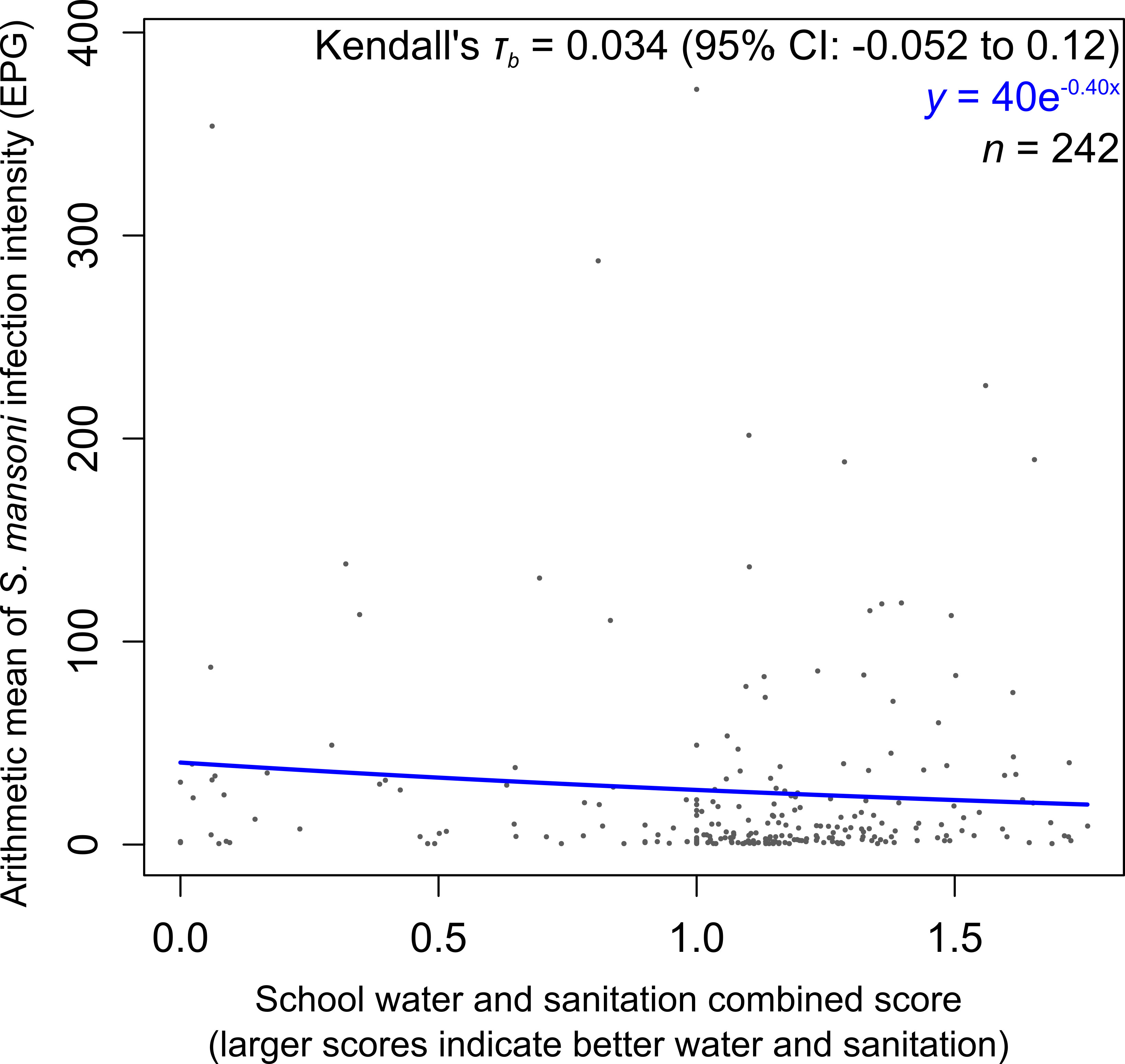

Supplement: S3 Fig — Kendall’s τb statistics, the equation of the least-squares line of best fit, and the number of included schools, are presented in the upper-right corner. (JPG) [file pntd.0004515.s003.jpg]

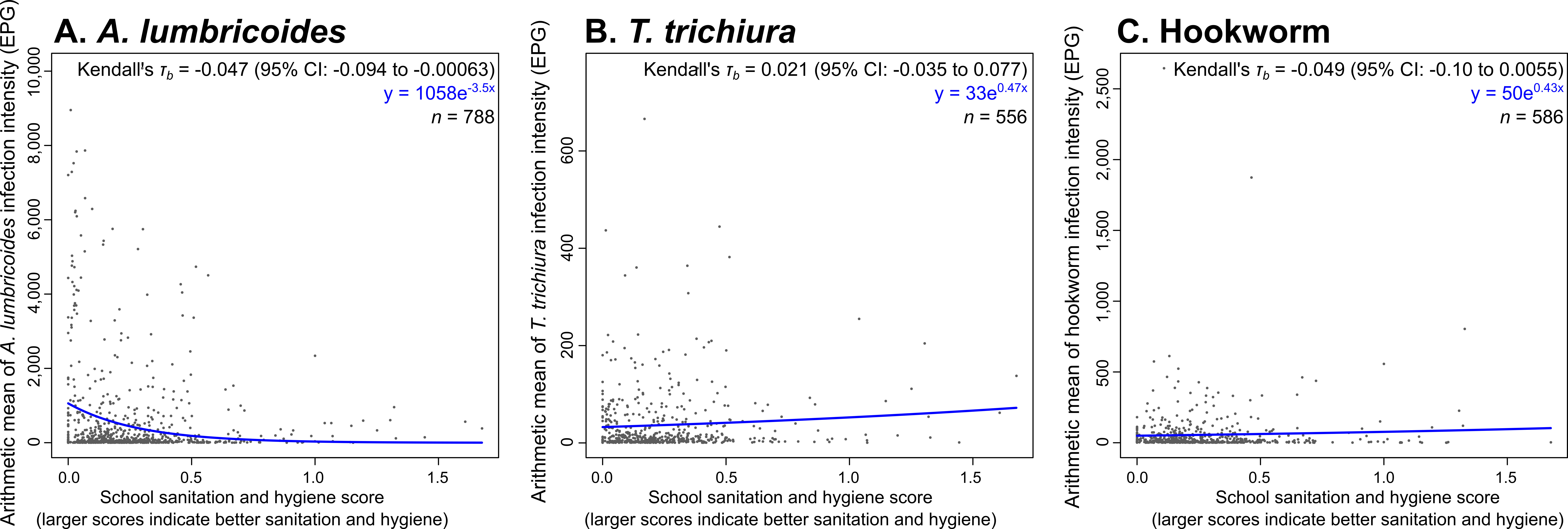

Supplement: S4 Fig — The Kendall’s τb statistics, least-squares best fit line equation, and sample size are presented in the upper-right corner of each graph. (JPG) [file pntd.0004515.s004.jpg]
